# Supplementary material for: The role of partner influence in contraceptive adoption, discontinuation, and switching in a nationally representative cohort of Ugandan women
Source: PLoS One. 2021 Jan 12;16(1):e0238662. doi: 10.1371/journal.pone.0238662 (PMC7802956; doi:10.1371/journal.pone.0238662)
Supplement: S1 Table — (DOCX) [file pone.0238662.s001.docx]

**S1 Table. Comparison of baseline and follow-up samples with and without loss-to-follow-up weighting**

|  | Unweighted | | | Weighted | | |
| --- | --- | --- | --- | --- | --- | --- |
|  | Round 6 Only  (%) | Round 6 Follow-Up  (%) | p-value | Round 6 Only  (%) | Round 6 Follow-Up  (%) | p-value |
| Age (mean) | 27.94 | 29.76 | <0.001 | 27.85 | 27.91 | 0.74 |
| Married or in-union | 63.27 | 72.56 | <0.001 | 63.83 | 64.19 | 0.75 |
| Urban | 27.22 | 20.87 | <0.001 | 22.38 | 23.08 | 0.65 |
| Ever given birth | 73.90 | 82.25 | <0.001 | 74.39 | 74.91 | 0.69 |
| Education |  |  |  |  |  |  |
| None | 12.23 | 14.70 | <0.001 | 9.49 | 9.25 | 0.49 |
| Primary | 53.41 | 56.95 | <0.001 | 54.77 | 54.46 | 0.76 |
| O-level | 24.99 | 21.49 | <0.001 | 26.19 | 26.38 | 0.86 |
| A-level + | 9.38 | 6.86 | <0.001 | 9.55 | 9.91 | 0.56 |
| Delay pregnancy 2+ years | 75.51 | 76.77 | 0.22 | 76.85 | 77.87 | 0.19 |
| Using modern method | 28.15 | 29.44 | 0.25 | 30.28 | 30.07 | 0.79 |
| Partner support now | 83.19 | 84.94 | <0.001 | 83.06 | 85.15 | 0.12 |
| Partner support future | 76.68 | 71.62 | <0.001 | 76.38 | 74.49 | 0.32 |
